# Supplementary material for: Deciphering the phase transition-induced ultrahigh piezoresponse in (K,Na)NbO3-based piezoceramics
Source: Nat Commun. 2022 Jun 15;13:3434. doi: 10.1038/s41467-022-31158-x (PMC9197837; doi:10.1038/s41467-022-31158-x)
Supplement: Supplementary file 1 — Supplementary Information [file 41467_2022_31158_MOESM1_ESM.pdf]

Supplementary information of

Deciphering the phase transition-induced ultrahigh piezoresponse in $(K,Na)NbO_3$-based piezoceramics

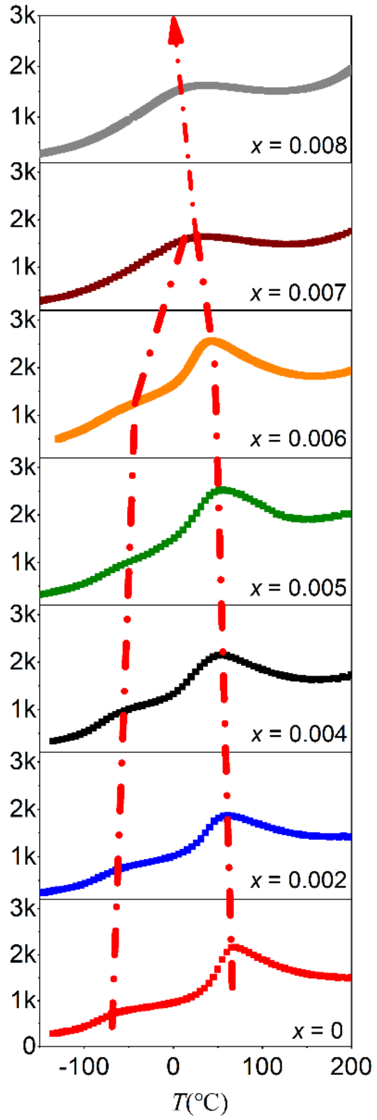

Supplementary Fig. 1. Temperature-dependent dielectric permittivity of all investigated KNN compositions $(0.97\text{-}x)K_{0.50}Na_{0.50}Nb_{0.965}Sb_{0.035}O_3\text{-}0.03(Bi_{0.5}Na_{0.5})_{0.9}(Ga_{0.5}Li_{0.5})_{0.1}ZrO_3\text{-}xBiFeO_3$ (x = 0, 0.002, 0.004, 0.005, 0.006, 0.007 and 0.008). Two dielectric anomalies observed for composition $x = 0$ at low and high temperatures represent the rhombohedral-orthorhombic phase transition temperature $T_{R\text{-}O}$ and the orthorhombic-tetragonal phase transition temperature $T_{O\text{-}T}$. $T_{R\text{-}O}$ increases, while $T_{O\text{-}T}$ decreases with increasing x.

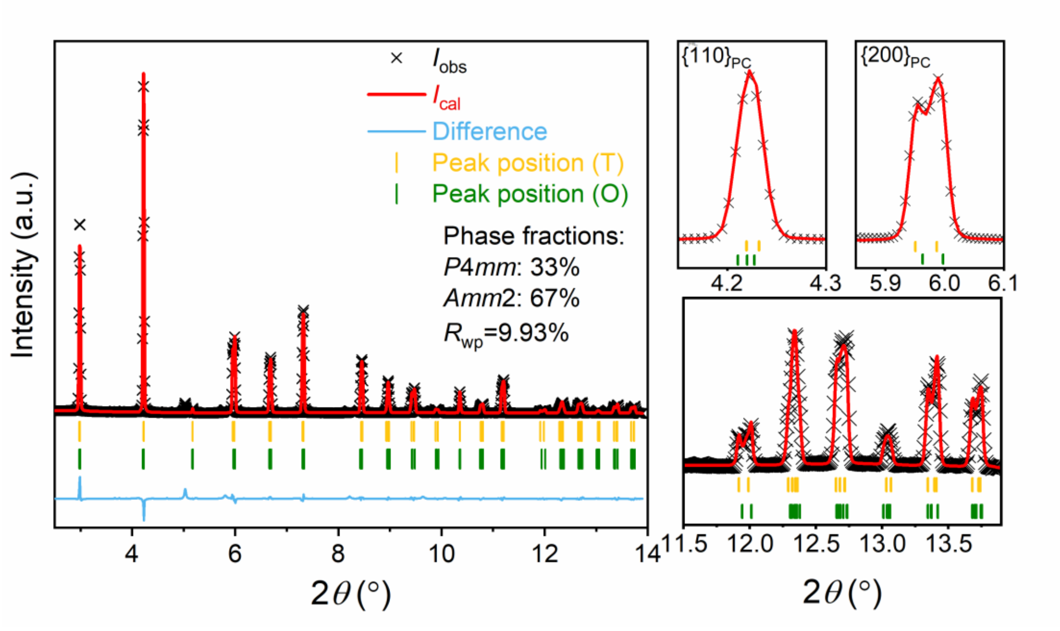

Supplementary Fig. 2. Rietveld refinements of the high-energy X-ray diffraction pattern of composition $x = 0.006$. An enlarged view of the $\{110\}_{PC}$, $\{200\}_{PC}$, and high-angle reflections is also highlighted. The full-range diffractograms were fitted using the Rietveld refinement method using GSAS software package. The starting parameters of the *Amm*2 and *P*4*mm* models for the refinement were taken from the X-ray and neutron powder diffraction data refinements of Orayech *et al.* [1] and Ishizawa *et al.* [2], respectively. The atom positions and the isotropic temperature factors, U_{iso}, were constrained for atoms sharing the same site. The A-site atom position was set as the reference and fixed at the origin. The wavelength of the used X-rays is 0.20714 Å.

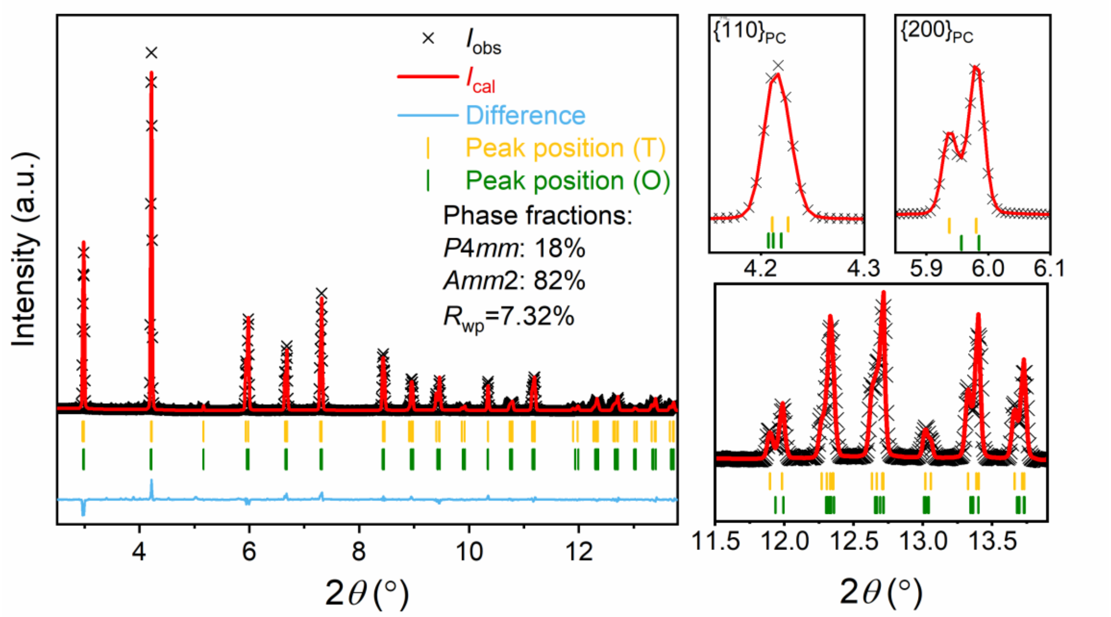

Supplementary Fig. 3. Rietveld refinements of the high-energy X-ray diffraction pattern of composition $x = 0.002$. Both enlarged views of the $\{110\}_{PC}$, $\{200\}_{PC}$, as well as high-angle reflections are provided. The wavelength of the used X-rays is 0.20714 Å.

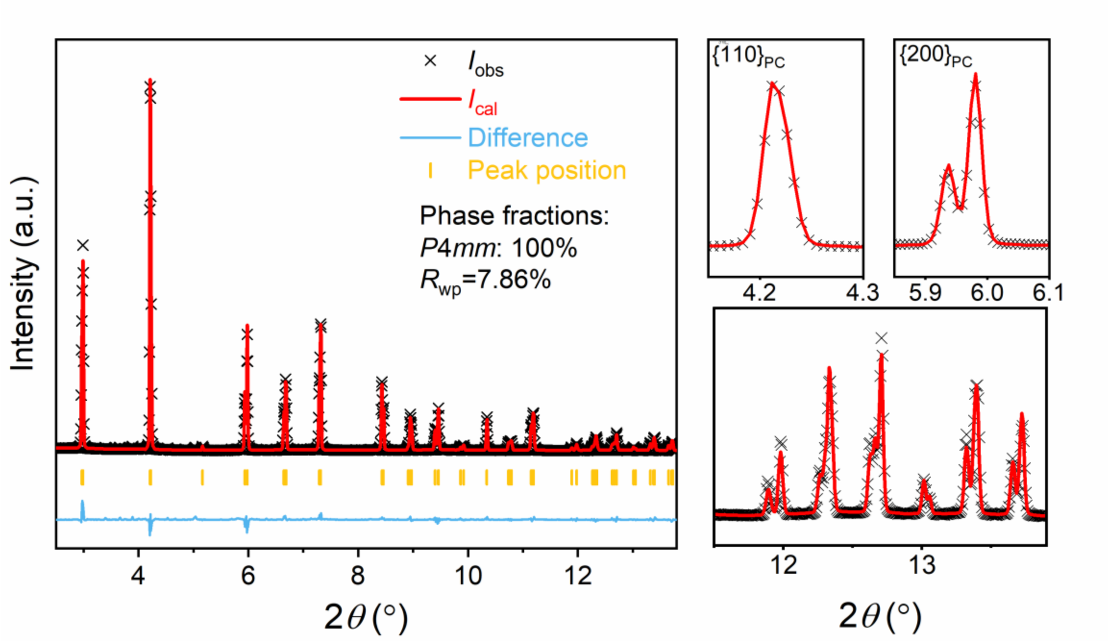

Supplementary Fig. 4. Rietveld refinements of the high-energy X-ray diffraction pattern of the $x = 0.008$ composition. Enlarged view of the $\{110\}_{PC}$, $\{200\}_{PC}$, and high-angle reflections is depicted. The pattern is fitted using a single *P*4*mm* model. The wavelength of the used X-rays is 0.20714 Å.

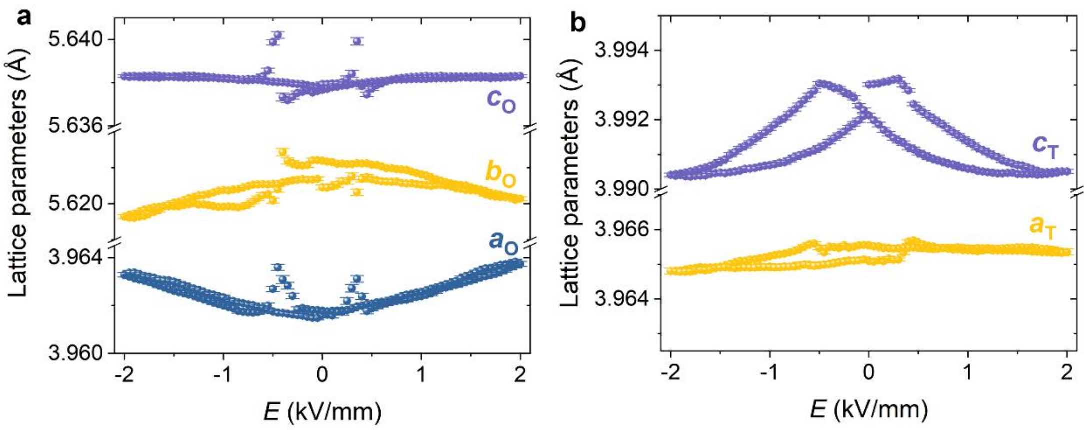

Supplementary Fig. 5. Evolution of lattice parameters of coexisting **a** orthorhombic and **b** tetragonal phases for the composition $x = 0.006$ as a function of bipolar electric field, calculated by the method STRAP (Strain, Texture, and Rietveld Analysis for Piezoceramics) [3].

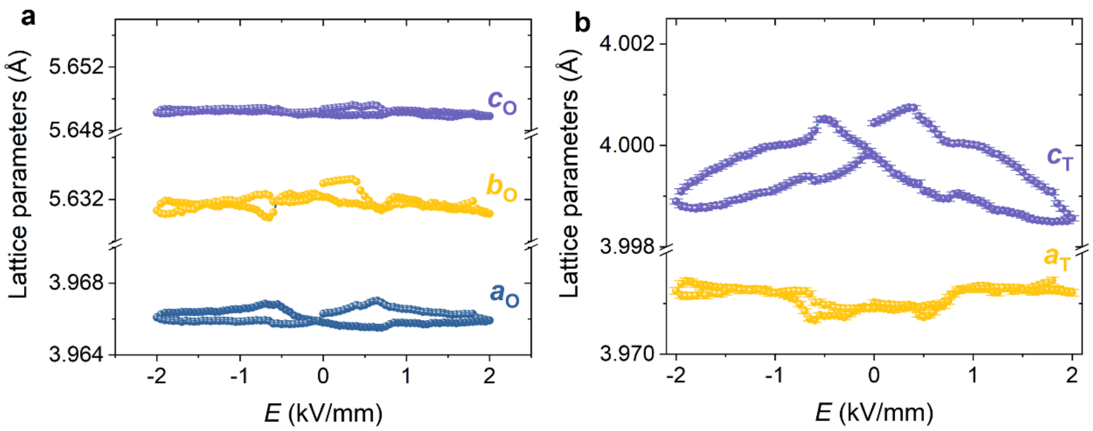

Supplementary Fig. 6. Evolution of lattice parameters of coexisting **a** orthorhombic and **b** tetragonal phases for the composition $x = 0.002$ as a function of bipolar electric field, calculated by the method STRAP (Strain, Texture, and Rietveld Analysis for Piezoceramics) [3].

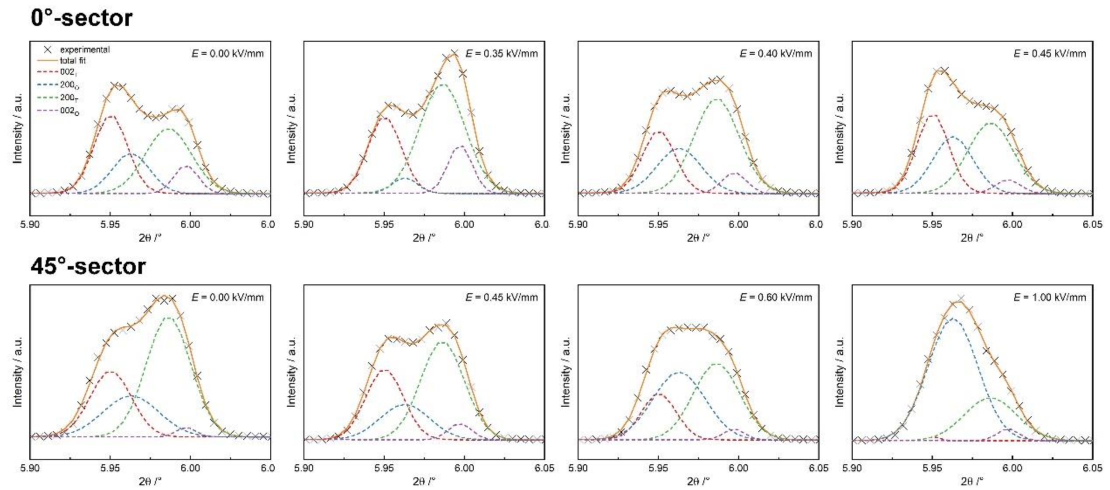

Supplementary Fig. 7. Representative fitting results of the {200} reflections of the composition x=0.006 in the 0°-sector and 45°-sector. The four peaks from low angle to high angle correspond to the 002_T, 200_O, 200_T, and 002_O reflections. The peak positions were determined from the Rietveld refinement of the x = 0.006 composition and were fixed for the fitting at different fields.

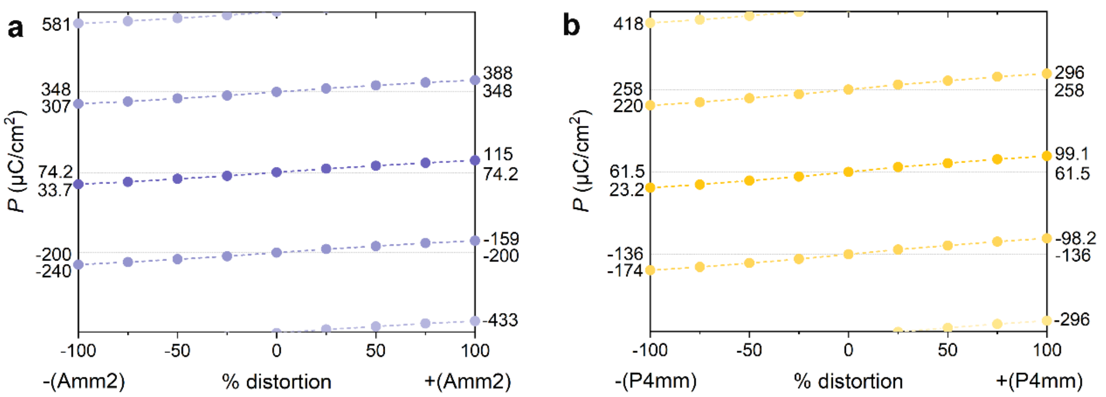

Supplementary Fig. 8 Electric polarization along the adiabatic paths from a the original *Amm*2 structure and b the *P*4*mm* structure through the centrosymmetric cubic structure to the corresponding inverted −(*Amm*2) and −(*P*4*mm*) structures.

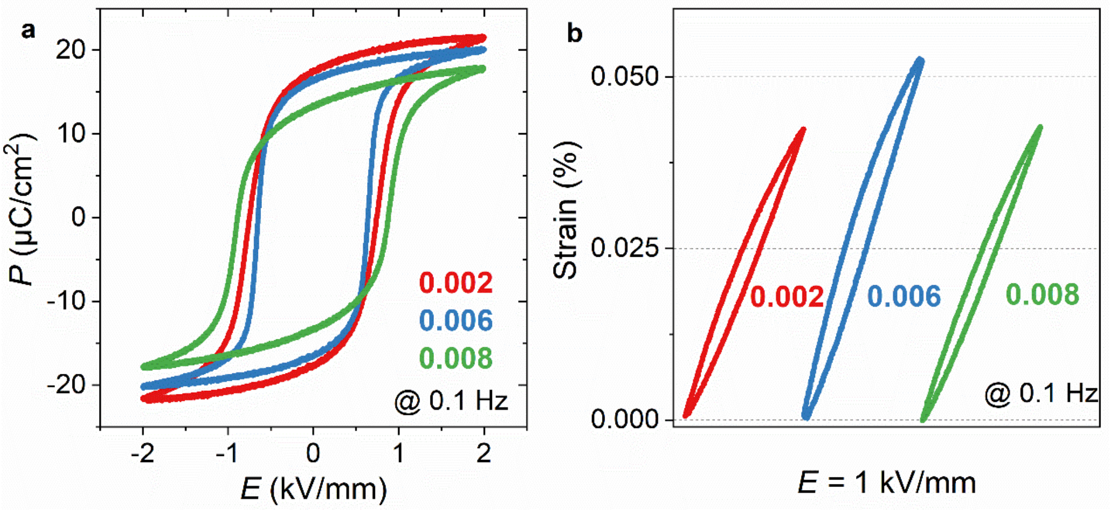

Supplementary Fig. 9 (a) Polarization-electric field (*P*-*E*) hysteresis loops and (b) unipolar strain-electric field (*S*-*E*) hysteresis loops of compositions x = 0.002, 0.006, and 0.008, measured at a frequency of 0.1 Hz with a maximum field of 2 kV/mm in (a) and 1 kV/mm in (b).

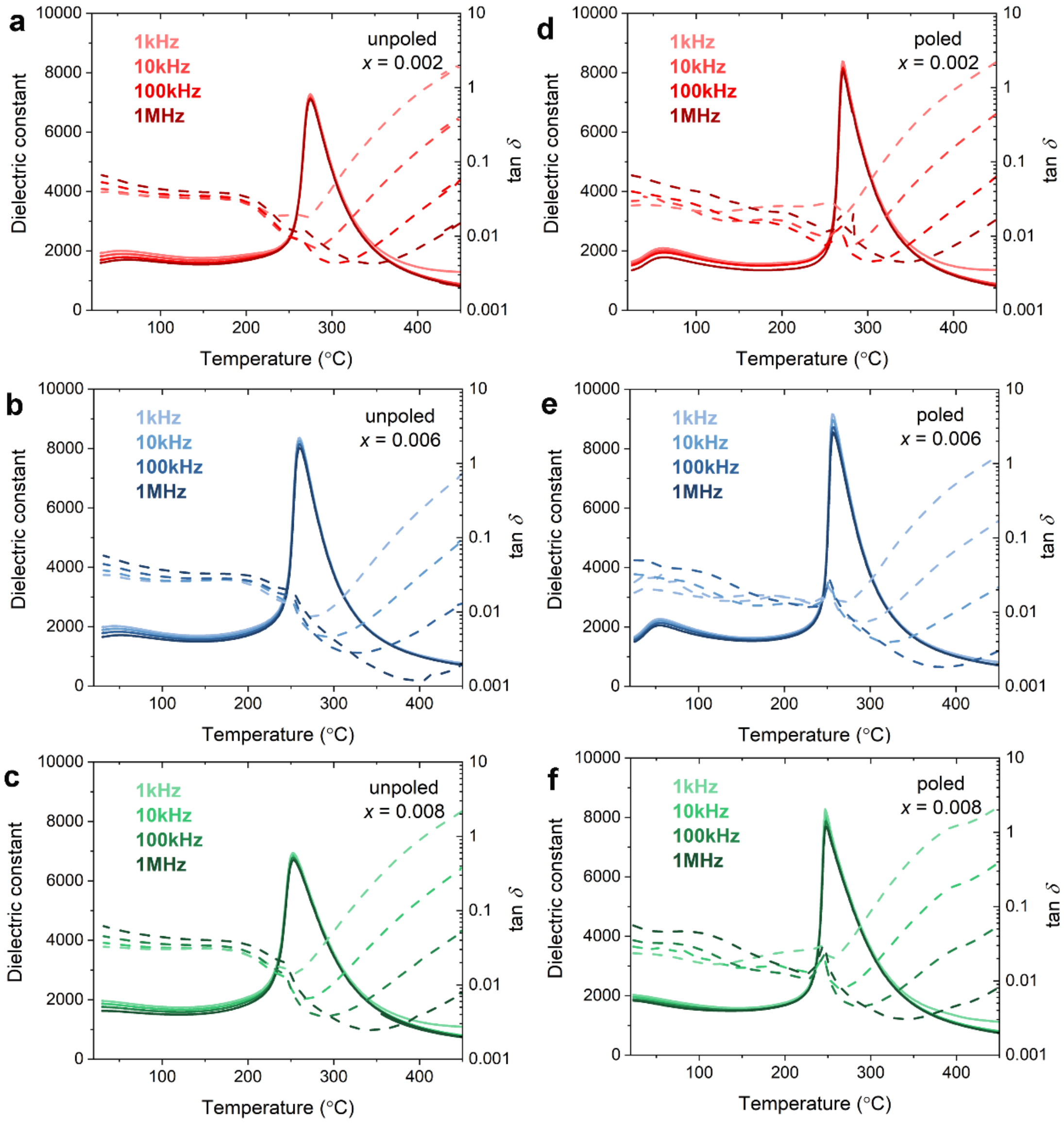

Supplementary Fig. 10 Temperature dependence of dielectric constant and loss tangent, tan δ, of compositions (a) $x = 0.002$, (b) 0.006, and (c) 0.008 in the unpoled state and (d) $x = 0.002$, (e) 0.006, and (f) 0.008 in the poled state, quantified at a frequency of 1 kHz−1 MHz.

	$x = 0.002$	$x = 0.006$	$x = 0.008$
R_{wp}	7.32	9.93	7.86
R_{exp}	4.04	2.86	4.25
GOF (χ^2)	1.81	3.47	1.85
*Amm*2			
Percentage (%)	81.76(7)	67.28(9)	-
a (Å)	3.96995(27)	3.96590(13)	-
b (Å)	5.6424(11)	5.6328(4)	-
c (Å)	5.6413(13)	5.64550(27)	-
A-site *x*	0	0	-
A-site *y*	0	0	-
A-site *z*	0.0194(1)	0.0419(7)	-
B-site *x*	0.5	0.5	-
B-site *y*	0	0	-
B-site *z*	0.4853(3)	0.5165(3)	-
O(1) *x*	0	0	-
O(1) *y*	0	0	-
O(1) *z*	0.5560(6)	0.4592(1)	-
O(2) *x*	0.5	0.5	-
O(2) *y*	0.7925(3)	0.7939(7)	-
O(2) *z*	0.1759(0)	0.2402(8)	-
A-site U_{iso}	0.0152(3)	0.0273(3)	-
B-site U_{iso}	0.0071(5)	0.0068(9)	-
O-site U_{iso}	0.0136(1)	0.023(1)	-
*P*4*mm*			
Percentage (%)	18.23(3)	32.71(1)	100
a (Å)	3.97264(6)	3.97166(15)	3.94450(33)
c (Å)	4.00191(10)	3.99766(19)	4.00342(6)
A-site *x*	0	0	0
A-site *y*	0	0	0
A-site *z*	0	0	0
B-site *x*	0.5	0.5	0.5
B-site *y*	0.5	0.5	0.5
B-site *z*	0.4850(2)	0.4674(30)	0.4823(1)
O(1) *x*	0.5	0.5	0.5
O(1) *y*	0.5	0.5	0.5
O(1) *z*	−0.0260(7)	−0.0002(1)	−0.0494(3)
O(2) *x*	0.5	0.5	0.5
O(2) *y*	0	0	0
O(2) *z*	0.4565(4)	0.4990(3)	0.4566(9)
A-site U_{iso}	0.0171(1)	0.0261(8)	0.0253(9)

B-site U_{iso}	0.0055(7)	0.0071(1)	0.0063(5)
O-site U_{iso}	0.010(7)	0.021(6)	0.0177(10)

Supplementary Table 1. Results of Rietveld refinement for the compositions x = 0.002, x = 0.006, and x = 0.008. Refined structural parameters of compositions x = 0.002 and x = 0.006 with *Amm*2 and *P*4*mm* models and refined structural parameters of composition x = 0.008 with *P*4*mm* model.

Reference

[1] Orayech, B.; Faik, A.; López, G. A.; Fabelo, O.; Igartua, J. M. Mode-crystallography analysis of the crystal structures and the low- and high-temperature phase transitions in $Na_{0.5}K_{0.5}NbO_3$. Journal of Applied Crystallography, 2015, 48(2), 318-333.

[2] Ishizawa, Nobuo; Wang, Jun; Sakakura, Terutoshi; Inagaki, Yumi; Kakimoto, Ken-ichi. Structural evolution of $Na_{0.5}K_{0.5}NbO_3$ at high temperatures. Journal of Solid State Chemistry, 2010, 183(11), 2731-2738.

[3] Hinterstein, M., Lee, K. Y., Esslinger, S., Glaum, J., Studer, A. J., Hoffman, M., & Hoffmann, M. J. Determining fundamental properties from diffraction: Electric field induced strain and piezoelectric coefficient. Physical Review B, 2019, 99(17), 174107.
