# Supplementary material for: Deciphering the phase transition-induced ultrahigh piezoresponse in (K,Na)NbO3-based piezoceramics
Source: Nat Commun. 2022 Jun 15;13:3434. doi: 10.1038/s41467-022-31158-x (PMC9197837; doi:10.1038/s41467-022-31158-x)
Supplement: Supplementary file 3 — Reporting Summary [file 41467_2022_31158_MOESM3_ESM.pdf]

nature portfolio

Corresponding author(s): Ke Wang

Last updated by author(s): Apr 14, 2022

Reporting Summary

Nature Portfolio wishes to improve the reproducibility of the work that we publish. This form provides structure for consistency and transparency in reporting. For further information on Nature Portfolio policies, see our Editorial Policies and the Editorial Policy Checklist.

Statistics

For all statistical analyses, confirm that the following items are present in the figure legend, table legend, main text, or Methods section.

n/a	Confirmed	
☐	☒	The exact sample size (n) for each experimental group/condition, given as a discrete number and unit of measurement
☐	☒	A statement on whether measurements were taken from distinct samples or whether the same sample was measured repeatedly
☒	☐	The statistical test(s) used AND whether they are one- or two-sided<br>*Only common tests should be described solely by name; describe more complex techniques in the Methods section.*
☒	☐	A description of all covariates tested
☒	☐	A description of any assumptions or corrections, such as tests of normality and adjustment for multiple comparisons
☒	☐	A full description of the statistical parameters including central tendency (e.g. means) or other basic estimates (e.g. regression coefficient) AND variation (e.g. standard deviation) or associated estimates of uncertainty (e.g. confidence intervals)
☒	☐	For null hypothesis testing, the test statistic (e.g. F, t, r) with confidence intervals, effect sizes, degrees of freedom and P value noted<br>*Give P values as exact values whenever suitable.*
☒	☐	For Bayesian analysis, information on the choice of priors and Markov chain Monte Carlo settings
☒	☐	For hierarchical and complex designs, identification of the appropriate level for tests and full reporting of outcomes
☒	☐	Estimates of effect sizes (e.g. Cohen's d, Pearson's r), indicating how they were calculated

Our web collection on statistics for biologists contains articles on many of the points above.

Software and code

Policy information about availability of computer code

Data collection	Python was used to control the collection of in situ synchrotron data, and labview was used to measure the electrical properties.
Data analysis	Matlab and Fit2D used to analyze the synchrotron data, and origin was used to process the electrical data.

For manuscripts utilizing custom algorithms or software that are central to the research but not yet described in published literature, software must be made available to editors and reviewers. We strongly encourage code deposition in a community repository (e.g. GitHub). See the Nature Portfolio guidelines for submitting code & software for further information.

Data

Policy information about availability of data

All manuscripts must include a data availability statement. This statement should provide the following information, where applicable:

- Accession codes, unique identifiers, or web links for publicly available datasets
- A description of any restrictions on data availability
- For clinical datasets or third party data, please ensure that the statement adheres to our policy

The relevant data sets generated during and/or analyzed during the current study are available from the first authors and corresponding authors on reasonable request.

Field-specific reporting

Please select the one below that is the best fit for your research. If you are not sure, read the appropriate sections before making your selection.

☐ Life sciences ☐ Behavioural & social sciences ☒ Ecological, evolutionary & environmental sciences

For a reference copy of the document with all sections, see nature.com/documents/nr-reporting-summary-flat.pdf

Ecological, evolutionary & environmental sciences study design

All studies must disclose on these points even when the disclosure is negative.

Study description	We developed a kind of high-performance piezoceramics and reveal the underlying mechanisms of the structure-property relationship. The properties and structures of the reported materials are based on reliable experimental data that can be reproduced.
Research sample	Functional ceramic samples with the nominal compositions (0.97-x)K0.50Na0.50Nb0.965Sb0.035O3-0.03(Bi0.5Na0.5)0.9(Ga0.5Li0.5)0.1ZrO3-xBiFeO3 (x = 0, 0.002, 0.004, 0.005, 0.006, 0.007 and 0.008) were prepared based on the conventional solid-state reaction methods. They were developed based on the the previous studies and judicious compositional design. More than 10 samples for each composition were sintered.
Sampling strategy	Once the optimized sintering conditions were found out, a series of electrical and structural studies were performed on different samples for each investigated composition. The reported results are based on the fact that the result can be reproduced on different samples.
Data collection	M.Z. and K.W. conceived the idea of this work. B.W. prepared the (K,Na)NbO3-based ce-ramic materials with the support from J.M. C.S. and M.D. performed the DFT calculations under the supervision of H.Z. and P.L. M.H. and MZ. performed the in situ synchrotron XRD measurements with the assistance of L.S. and A.S. M.Z., C.Z., and M.H. analyzed the in situ synchrotron data and discussed in detail with N. Z. F.Y. and Q. Y. are responsible for the TEM investigation and received helpful assistance from H.N. and D.W. for the processing of the TEM data. M.Z. and B.W. conducted all electrical measurements and L.F. finished the latent heat measurement. M.Z. analyzed the structure-property relationship with the help of C.Z. M.Z. drafted the first version of the manuscript and all authors participated in the writing of the paper. K.W. and M.Z. guided the projects. K.W., J.L. and J.R. provided financial and technical support for the accomplishment of this work and provided helpful suggestions.
Timing and spatial scale	Not applicable to our study, because the functional properties and structures of the investigated piezoceramics usually do not decay with time.
Data exclusions	No data were excluded.
Reproducibility	In general, once the optimized processing conditions are found out and fixed, the structures and functional properties of piezoceramics can be well reproduced. The ceramics samples were prepared in Chengdu, China and basic electrical characterizations were initially performed in Chengdu and Beijing, China. Detailed electrical measurements were done in Darmstadt, Germany and the electrical properties could be well reproduced. Detailed structural studies were performed in Hamburg, Germany and could nicely support the macroscopic properties obtained elsewhere. All attempts to repeat the experiments were successful.
Randomization	Not applicable to our study, because once functional piezoceramics with the optimized conditions were synthesized and sintered, the reproducibility of the macroscopic properties and structures are very good. Each ceramic sample from the same batch usually exhibits very similar properties. No randomization is needed.
Blinding	Not applicable to our study, because once functional piezoceramics with the optimized conditions were synthesized and sintered, the reproducibility of the macroscopic properties and structures are very good. Each ceramic sample from the same batch usually exhibits very similar properties. Blinding is not necessary.

Did the study involve field work? ☐ Yes ☒ No

Reporting for specific materials, systems and methods

We require information from authors about some types of materials, experimental systems and methods used in many studies. Here, indicate whether each material, system or method listed is relevant to your study. If you are not sure if a list item applies to your research, read the appropriate section before selecting a response.

Materials & experimental systems

n/a	Involved in the study
x	Antibodies
x	Eukaryotic cell lines
x	Palaeontology and archaeology
x	Animals and other organisms
x	Human research participants
x	Clinical data
x	Dual use research of concern

Methods

n/a	Involved in the study
x	ChIP-seq
x	Flow cytometry
x	MRI-based neuroimaging
